# Multiple primary malignancies and subtle mucocutaneous lesions associated with a novel *PTEN *gene mutation in a patient with Cowden syndrome: Case report

**DOI:** 10.1186/1471-2350-12-38

**Published:** 2011-03-15

**Authors:** Peter Vasovčák, Mária Šenkeříková, Jana Hatlová, Anna Křepelová

**Affiliations:** 1 Department of Biology and Medical Genetics, Charles University 2nd Faculty of Medicine and University Hospital Motol, Prague, Czech Republic; 2 Department of Medical Genetics, University Hospital, Hradec Králové, Czech Republic; 3 Fingerland's Department of Pathology, Faculty of Medicine, Hradec Králové, Czech Republic

## Abstract

**Background:**

Cowden syndrome (CS) is a cancer predisposition syndrome associated with increased risk of breast, thyroid, and endometrial cancers, and is characterized by development of benign mucocutaneous lesions.

**Case presentation:**

Here we report on a 58-year-old woman with multiple primary malignancies and subtle mucocutaneous lesions such as small polyps and wart-like papulas. Over a period of 23 years, she developed various malignant neoplasms including thyroid, ovarian, stomach, and colon carcinomas, and a benign meningioma. Direct sequencing analysis of the *PTEN *gene revealed a novel germline mutation (c.438delT, p.Leu146X).

**Conclusion:**

This case demonstrates that Cowden syndrome is a multi-system disease that can result in the development of multiple malignant and benign tumors.

## Background

Cowden syndrome (CS, OMIM 158350) is an autosomal dominant disorder characterized by multiple hamartomas, which develop in the skin, thyroid gland, breast, gastrointestinal tract, and brain[1]. Germline mutations in the *PTEN *(phosphatase and tensin homolog deleted on chromosome ten) gene have been found in 80% of patients with CS[2]. The *PTEN *gene encodes a dual-specificity protein and lipid phosphatase that regulates the phosphoinositol-3-kinase/Akt signal pathway which can result in cell cycle arrest in the G1-phase and apoptosis[3]. PTEN can directly or indirectly dephosphorylate focal adhesion kinase (FAK), resulting in inhibition of cell migration and cell spreading[3-5].

Diagnosing CS remains a challenge due to the variations in its clinical presentation. A recent molecular-genetic study estimated that the incidence of CS was 1/200 000, although the actual incidence is likely to be higher[6]. The pathognomonic features of CS include mucocutaneous wart-like lesions, which are present in 99% of affected individuals before the age of 30 years[3]. The other most commonly reported manifestations are breast carcinomas, thyroid abnormalities, macrocephaly, hamartomatous polyps and mental retardation [7,8]. In women with CS, the lifetime risk of breast cancer is estimated to be 25-50%, compared to the general female population risk of approximately 11%. The lifetime risk of epithelial thyroid cancer can be as high as 10% in both males and females with CS. Follicular carcinoma is the most common CS-associated thyroid cancer, although papillary carcinomas have also been observed [9,10].

The present report describes the identification of a novel *PTEN *germline mutation in a female CS patient who developed multiple primary tumors and subtle skin lesions.

## Case presentation

A 55-year-old woman suspected to have CS was referred to the Department of Biology and Medical Genetics, University Hospital Motol, Prague, for *PTEN *gene analysis to confirm the diagnosis.

**Figure 1:**
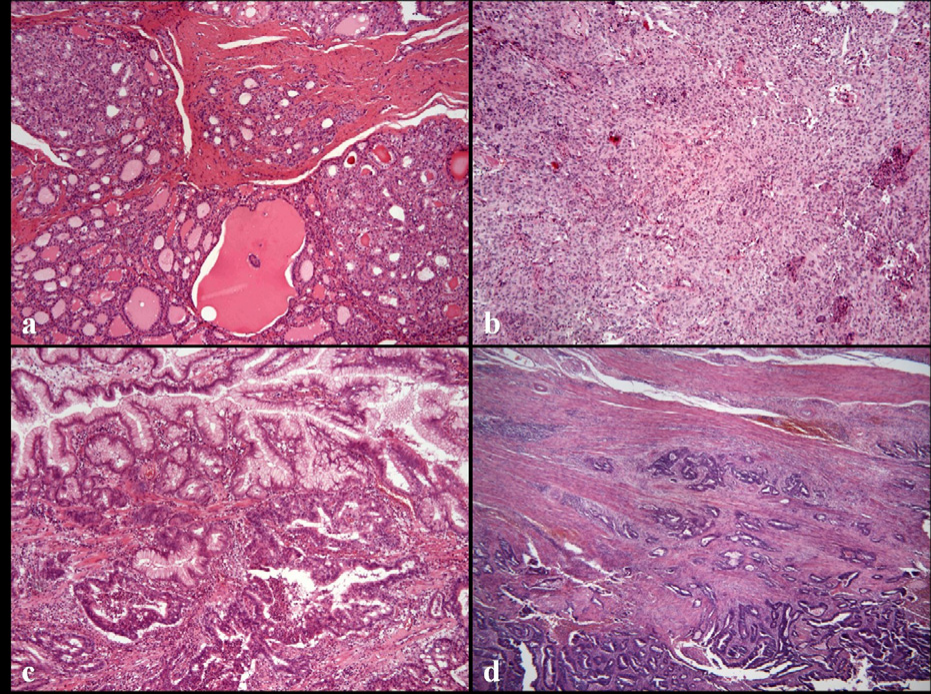
**Histopathologic examination of the CS patient**. a) Minimally invasive follicular carcinoma of the thyroid gland (H&E ×100). b) Meningotheliomatous meningioma (H&E ×100). c) Gastric hyperplastic polyp with adenocarcinoma (H&E ×200). d) Tubular adenocarcinoma of the large bowel (H&E ×100).

Her medical history was unremarkable until she developed a goiter at 34 years of age. At 45 years of age, she underwent a strumectomy. A histopathological examination confirmed a macrofollicular adenoma in the left thyroid lobe and an encapsulated thyroid tumor in the right thyroid lobe. At 54 years of age she underwent a total thyroidectomy due to a follicular carcinoma (Figure 1a).

At 43 years of age she underwent a hysterectomy for myomatosis and adnexectomy due to an adenopapilocarcinoma of the left ovary.

At the age of 49 years, the patient was admitted to hospital with epileptic paroxysms. A CT scan of the brain showed a left frontal lesion, which was the cause of the paroxysms. The lesion was surgically removed and histopathologically classified as a benign meningioma (Figure 1b). At 53 and 57 years of age, she was again surgically treated for meningioma recurrence and a steadily deteriorating condition. Despite those surgical interventions, there was a progressive decline in cognitive functions and memory.

The initial brain tumor finding together with the observed cachexia led to the suspicion of metastasis from an unknown primary cancer. Careful endoscopic examination revealed hundreds of polyps in the stomach, duodenum and colon. There were numerous mostly hyperplastic and hamartomatous polyps up to 10 mm in diameter in the stomach. Smaller and multiple sessile polyps were found in the duodenum. The esophagus was free of any polyps. Along the entire length of the colon were multiple histopathologically confirmed hyperplastic and inflammatory polyps as well as tubulovillous or villous adenomas of 4-10 mm in diameter, with low-grade dysplasia.

At the age of 55 years, the patient was examined by a clinical geneticist (MS) who observed macrocephaly, a fissured tongue and a polyp of 0.5 cm in diameter in the dorsal part of the oral cavity. A few wart-like papules on the forearms and chest were classified as senile verrucoid lesions. No other mucocutaneous lesions were found on the face or oral mucosa. The patient's mother, whose medical history was unknown to us, died of colon cancer at the age of 56 years. No other members of the family have had signs of CS or any cancer. The patient had one child who was negatively tested for mutations in the *PTEN *gene.

Several biopsies were performed throughout the gastrointestinal (GI) tract over a 9-year period, and none showed evidence of malignant lesions. Then at 57 years of age, histopathological examination of biopsy material from a hyperplastic polyp in the stomach revealed a well-differentiated intramucosal adenocarcinoma of 8 mm in diameter (Figure 1c). One year after the gastric cancer had been detected, the patient developed an aggressively growing synchronous adenocarcinoma of the anorectum (T3N0M0) and the sigmoid colon (T3N0M0) (Figure 1d). Abdominoperineal surgery (i.e., the Miles' operation) was performed. However, the patient died at post-operative 1 month due to unmanageable complications.

## Methods

This study was approved by the Ethics Committee of the University Hospital Motol, Prague, according to the Helsinki Principles. After receiving written informed consent, the genomic DNA of the patient was isolated from blood leukocytes using a Genomic DNA Purification Kit (Gentra Systems, Minneapolis, MI, USA) according to the manufacturer's guide. The genomic DNA was amplified using intronic primers (sequences are available upon request) flanking the nine exons and a promoter region of the *PTEN *gene[11]. The PCR products were purified using a SureClean PCR purification kit (Bioline, London, UK). Bidirectional cycle sequencing of the PCR products was performed using a BigDye Terminator v3.1 Cycle Sequencing kit and an ABI 3130 Genetic Analyzer (both from Applied Biosystems, Foster City, CA, USA).

## Results and Discussion

**Figure 2:**
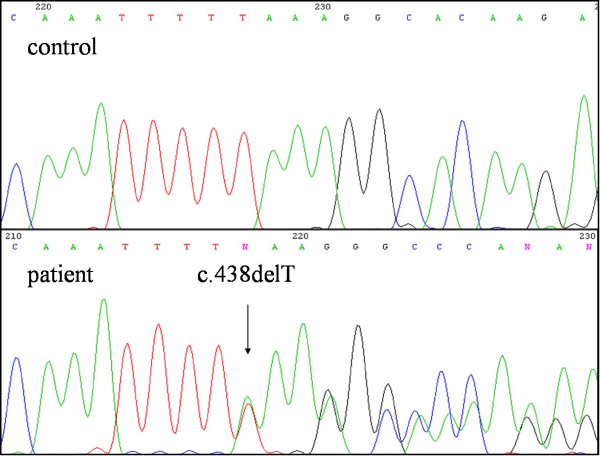
**DNA sequencing electropherograms**. Upper panel: Electropherogram of the *PTEN *gene, exon 5, from a healthy control. Lower panel: Electropherogram of the *PTEN *gene, exon 5, from the Cowden syndrome patient. Note the heterozygous deletion mutation at c.438delT (arrow).

Examination of the genomic DNA revealed a novel heterozygous mutation, c.438delT, in exon 5 (Figure 2). That mutation is predicted to lead to a frameshift that results in formation of a premature stop codon (p.Leu146X) for PTEN protein translation. The mutation is considered to be pathogenic and causative for CS disease.

More than 80% of patients who strictly meet the International Cowden Consortium criteria are found to harbor a *PTEN *mutation[12]. Mucocutaneous lesions are present in 99% of CS patients before the age of 30 years[3]. However, the present patient was not reported to have had mucocutaneous lesions until 55 years of age, when a targeted examination revealed subtle skin lesions. The only visible wart-like papules were found on her forearms. Such lesions are commonly found in the general population and could therefore have been overlooked or considered unremarkable.

The most common malignancies associated with CS are breast and thyroid carcinomas. Benign breast lesions (fibrocystic breast disease, fibroadenomas) and breast adenocarcinomas develop in about 76% and 25-50% of CS-affected females, respectively[3]. The average age of a breast cancer diagnosis in CS patients is between 38 and 46 years[13]. Marsh et al. observed an association between the presence of a *PTEN *mutation and malignant breast disease [12]. In our patient, a mammographic examination at 57 years of age showed no evidence of any breast lesions.

Benign thyroid lesions occur in as many as 60% and thyroid cancers in up to 10% of CS patients [3,9]. Our patient developed a follicular adenocarcinoma at 54 years of age, despite regular follow-up and treatment since her first admission to hospital due to a goiter at 34 years old.

Gynecological abnormalities are reported in 44% of females with CS [7]. Endometrial cancer is a major diagnostic criterion for CS, with an estimated frequency of 5-10%[7]. Ovarian tumors are rare in CS, with only one report documenting an ovarian dysgerminoma in a CS patient [14]. The hypothesis that a germline mutation of the *PTEN *gene was implicated in the formation of ovarian tumors in CS patients was supported by the identification of a somatic loss of heterozygosity (LOH) in the wild-type allele [14]. The present patient had myomatosis of the uterus and adenocarcinoma of the left ovary at the age of 43 years. Unfortunately, ovary tumor tissue was not available for LOH analysis.

Macrocephaly and Lhermitte-Duclos disease (dysplastic cerebellar gangliocytoma) are also major pathognomonic diagnostic criteria of CS[15]. Meningiomas (benign brain tumors) have also been described in CS patients, albeit rarely [16-19]. In our patient, the discovery of a meningioma launched an intensive and thorough follow-up which revealed additional and continually appearing symptoms. Benign brain lesions accompanied by epileptic paroxysms and a steadily deteriorating clinical condition were her main health problems.

Gastrointestinal polyps (mostly classified as hamartomatous, hyperplastic, inflammatory, juvenile, lymphomatous and adenomatous) are commonly reported in CS patients, with frequencies ranging between 35-85% and higher than 90% in Western and Japanese patients, respectively [9,20-24]. That difference in frequency is considered to reflect that GI examinations are more common in Japan[24]. In a study involving 126 CS patients, Marra et al., reported that of the 42 patients who underwent a complete GI examination, 36 (85%) had GI polyps. Non-adenomatous polyps that are generally asymptomatic may be more common in CS patients, especially when GI investigations are not part of the recommended surveillance for CS. Although an association between CS and GI cancer remains a topic of debate, reports describing malignant GI tumors in CS patients have been published [9,21-23,25-27]. Although non-adenomatous polyps have a weak or absent malignant potential, there is evidence that carcinomas may arise within non-adenomatous polyps [26,28, and the present study]. Marra et al. stated that adenomatous polyps represent approximately 25% of CS polyps, and have a higher malignant potential than non-adenomatous polyps[22]. During a 9-year follow-up period, the present patient developed numerous GI polyps with various histopathological features (hyperplastic, inflammatory, hamartomatous and adenomatous). Ultimately, three polyps (a hyperplastic polyp in the stomach, and adenomatous polyps in the rectum and the sigmoid colon) progressed to macroscopic carcinomas.

## Conclusion

Although breast and thyroid cancers are the predominant malignancies in CS patients, it should be emphasized that benign and/or malignant tumors may also develop in the GI tract from pre-existing polyps, and in the genitourinary tract or in the brain. Most of the studies that have examined cancer in CS patients do not report the lifetime risk of development of a malignant neoplasm because the patients are relatively young. We propose that meningiomas and GI tract cancers, albeit rare, should be a component of the definition of CS. Physicians who might encounter CS patients should be aware of the possible neurological and/or GI tract manifestations.

## Consent

Written informed consent was obtained from the patient for publication of this case report and for the use of the accompanying images. A copy of the written consent can be obtained from the Editor-in-Chief of this journal.

## Competing interests

The authors declare that they have no competing interests.

## Authors' contributions

PV carried out the molecular genetic study including the DNA sequencing, and drafted the manuscript. MS identified and diagnosed the patient. JH prepared, read and classified the histological samples. AK designed the study and revised the manuscript. All authors read and approved the final manuscript.

## Pre-publication history

The pre-publication history for this paper can be accessed here:

http://www.biomedcentral.com/1471-2350/12/38/prepub
